# The impact of perceived control and power on adolescents’ acceptance intention of intelligent online services

**DOI:** 10.3389/fpsyg.2022.1013436

**Published:** 2022-09-29

**Authors:** Ying Yan, Wenfang Fan, Bingjia Shao, Yuanyang Lei

**Affiliations:** ^1^Electronic Commerce School, Chongqing Business Vocational College, Chongqing, China; ^2^UCSI Graduate Business School, UCSI University, Kuala Lumpur, Malaysia; ^3^School of Economics and Business Administration, Chongqing University, Chongqing, China; ^4^Chongqing Key Laboratory of Logistics, School of Economics and Business Administration, Chongqing University, Chongqing, China

**Keywords:** adolescent, intelligent service, perceived control, perceived usefulness, sense of power, acceptance intention

## Abstract

A higher level of intelligence can improve adolescents’ interactions with intelligent online services, although overemphasizing intelligent online services may nullify their sense of autonomy and in turn affect their acceptance intention. Enterprises have therefore focused on the best ways through which to provide intelligent online services. Based on the technology acceptance model, this study constructs a theoretical model of the impact of perceived control and power on adolescents’ acceptance intention of intelligent services. Through a scenario experiment involving an intelligent online recommendation service, 195 participants were obtained to test the model. The results show that the adolescents’ perceived control affects their acceptance intention of intelligent online services through their perceived usefulness. The adolescents’ sense of power moderates the influence of perceived control on perceived usefulness. This study supplements the research on intelligent online services and provides a reference for online merchants seeking to design such service processes.

## Introduction

With the rapid development of the digital economy, the applications of artificial intelligence (AI) have been expanding into new and unexplored areas in recent years. According to McKinsey, AI is expected to increase global economic activity by US$13 trillion and account for 1.2% of global GDP growth over the next fifteen years (McKinsey Global Institute, 2018). In the context of the rapid development of AI, innovation in intelligent online services has gradually become a key way for enterprises to seek new opportunities and remain competitive. Accordingly, it has attracted the attention of marketing scholars and entrepreneurs, as effectively combining online services and AI technology can help enterprises handle data collection tasks, promote communication between buyers and sellers, improve the consumer experience while reducing risks, and significantly improve satisfaction (Ng and Wakenshaw, 2017). At present, intelligent online services have been pursued to effectuate upgrades in many fields and involve interfaces such as personalized AI consumer assistants and sentiment analysis systems to assist consumers in making purchasing decisions during travel (Hartmann et al., 2019; Singh et al., 2020).

With the continuous improvement of AI, emerging intelligent online services products reflect more personalized anthropomorphic attributes such as social interaction and emotional communication, which improve the degree of human–computer interaction and have been increasingly adopted by businesses as a result. However, with the increase in demand for consumer interaction, the non-human characteristics of AI technology also affect users’ behavior and decisions, which greatly reduces the utilization rate of intelligent services. For example, intelligent online services providers usually design their algorithms to obtain optimal results, which may lead to deviation in decision-making. Therefore, improving consumers’ acceptance of intelligent online services has become an increasingly widespread focus among software enterprises.

In terms of the actual consumer experience, intelligent online services usually provide precise and results-oriented services to consumers to help them achieve their goals. However, overemphasizing intelligent online services may largely destroy consumers’ sense of autonomy, reduce their perceived control, and directly affect their acceptance intention. Consumers’ perceived control in the service process will largely determine whether and the degree to which they accept intelligent services. In addition, consumers with different senses of power have varying levels of control over smart services. Perceived control refers to the extent to which consumers believe that they can control the service process and its results (Collier and Sherrell, 2010). When people’s freedom in their daily lives is threatened, they feel powerless because of their lack of control and may therefore begin to resist, both psychologically and behaviorally (Miller and Seligman, 1975). Therefore, it is crucial for online merchants to understand how to optimally design intelligent online services processes according to consumers’ sense of power.

Prior studies have focused on the interactive perspective of consumer perception in specific intelligent online services settings and investigate their characteristics and influence (Wirtz et al., 2018), their anthropomorphic behaviors (Adam et al., 2021), task-oriented types of intelligent online services (André et al., 2018; Castelo et al., 2019), and the effect of personalized interaction (Chattaraman et al., 2019; Huang and Rust, 2021a) on consumers’ attitudes (Verhagen et al., 2014; Fernandes and Oliveira, 2021), and behavioral intentions (Wirtz et al., 2018; McLean and Osei-Frimpong, 2019). There are few studies on the influence of consumer perceived control on the acceptance intention of intelligent services (Seo and Bernsen, 2016; Zhang et al., 2021). In addition, previous studies have mainly focused on positive attitudes towards intelligent online services and research on acceptance intentions among different age groups, such as the impact of adolescents’ active use of online services on adolescents’ growth (Liu et al., 2020; Li and Mora, 2022). However, from the perspective of marketing, with the emergence of intelligent services in more industries and fields, more and more users actively or unconsciously participate in them, and the influence of consumer perceived control plays a more significant role in consumers’ acceptance intentions. In addition, from the perspective of social interaction, previous studies have mainly examined the relationship between service providers and users while only considering the influence of utilitarianism and ignoring that of the sense of power. Therefore, it is of great theoretical and practical value to investigate the influence mechanism of perceived control on consumers’ intention to accept intelligent online services and the moderating effect of power sense from the perspective of the interaction between consumers and intelligent services.

This study focuses on the following research questions. First, does consumer-perceived control affect consumers’ acceptance intentions with regard to intelligent services? Second, what is the influence mechanism of consumer-perceived control on consumers’ acceptance intentions? Third, does sense of power have a moderating effect on this relationship? To answer these questions, this study develops a research model to investigate the impact of consumer-perceived control on consumers’ acceptance intention of intelligent online services and explore the moderating effect of their sense of power based on the technology acceptance model. This study supplements the research on intelligent online services and provides a reference for online merchants seeking to design and implement such processes.

## Hypothesis development

### Perceived control and acceptance intention of intelligent service

Intelligent services are a type of service innovation that incorporate the use of AI in an adaptive and autonomous system that directly communicates and interacts with customers and provides services (Wirtz et al., 2018). Unlike traditional human services, intelligent online services have unique intelligent characteristics in the dimensions of form (i.e., entity embedding, virtual interface), anthropomorphism (i.e., their degree of similarity to humans), and task orientation (i.e., computing prediction and emotional delivery tasks). Intelligent services offer a wide range of effects to consumers or business organizations through their large-scale storage and rapid analysis capabilities. Research has found that efficient and timely intelligent online services can improve consumers’ overall online experience (Paz and Delgado, 2020). The application of AI can promote the standardization of services, help marketers develop more personalized marketing strategies, and analyze consumers’ emotions in real-time to improve their attitudes (Ghasemaghaei, 2020; Huang and Rust, 2021a). However, previous research results indicate that there are potential problems in the practical application of intelligent services. For example, more and more data and decisions rely on real-time information in specific scenarios, while intelligent online services have limited access to information and may not be able to obtain sufficient information to make decisions in some cases (Huang and Rust, 2021b). Even in the presence of such data, it is possible that intelligent online services will make mistakes due to changing consumer behaviors (André et al., 2018).

Consumers’ perceived control can be influenced in the process of interacting with intelligent services. Perceived control is one of the important determinants of consumers’ behavioral intentions. Previous studies on perceived control are mainly based on the psychological resistance and planned behavior theories (Brehm, 1972; Fishbein and Ajzen, 2011). Perceived control is a necessary prerequisite for predicting intentions and behaviors (Conner and Armitage, 1998). In addition, in the consumer service recovery process, perceived control indirectly affects consumers’ overall satisfaction with the service (Chang, 2008).

Consumers’ acceptance intention refers to the their overall acceptance tendency with regard to intelligent online services technology. The technology acceptance model shows that the factors influencing consumers’ intention to accept technology are related to their performance expectations, effort level, social influence, optimism, and experience as well as its convenience and perceived cost (Venkatesh et al., 2012). Averill (1973) showed that selective perception has a positive impact on a person’s level of perceptual control, which can in turn influence their acceptance of intelligent online services (Reinders et al., 2008). In the process of consuming services, consumers’ perception of choice can amplify their perceived control and bring positive emotions, thereby increasing their intention to adopt technology (Hui and Bateson, 1991). Taking the perspective of consumer autonomy can help to understand the potential impact of consumer-perceived control on their acceptance intention of intelligent services. The key to the autonomous consumer experience is to make consumers aware of selective behaviors and minimize the constraints in their consumption decisions (André et al., 2018). Higher autonomy implies a higher level of perceived control, which may increase consumers’ well-being and influence their acceptance intentions towards new technologies. Therefore, this study assumes that with an improvement in consumers’ perceived level of control, they will have a greater sense of autonomy and thus be more willing to accept intelligent services. Therefore, we propose the following hypothesis:

*Hypothesis 1*: Perceived control positively affects consumers’ acceptance intention of intelligent services.

### Perceived control and perceived usefulness

According to the technology acceptance model, perceived usefulness refers to the degree to which individuals can improve their job performance by using technology (Davis, 1989). More specifically, it includes consumers’ subjective attitudes towards the usefulness of a technology after considering such factors as its image, job relevance, output quality, provability of results, and perceived ease of use as well as other subjective norms (Venkatesh et al., 2012). In the intelligent online services context, consumers can judge the usefulness of new technologies according to the full range of their perceptions, which form the basis of the perceived usefulness of intelligent services (Wirtz et al., 2018; Friedrich et al., 2019).

Perceived control can affect consumers’ risk perception with regard to autonomous service technologies (Lee and Allaway, 2002). Furthermore, this perceived risk can affect consumers’ judgment of the quality of the technology, which in turn significantly affects consumers’ perceptions of its usefulness (André et al., 2018). Eastlick et al. (2006) find that brand associations related to perceived control affect consumers’ trust in brands and thus their perception of the brands’ usefulness. In addition, perceived control can improve consumers’ tendency to actively acquire knowledge related to the service as well as the pleasure they derive from using it and increase its perceived ease of use and therefore its perceived usefulness (Collier and Sherrell, 2010; Zhang et al., 2021).

Cognitive dissonance theory can also be used to explain the influence of consumers’ perceived control on the perceived usefulness of intelligent services. In general, when individuals are limited in their choices, they feel devalued by the results they obtain (Pepitone and Festinger, 1959). Previous studies have found that when consumers’ decisions are restricted, they may have a persistently negative attitude towards service providers. In addition, Seo and Bernsen (2016), in studying the influence of perceived usefulness on consumers’ acceptance intentions, find that the influence of individuals’ constraints should be considered. Therefore, this study assumes that in the context of smart services, the higher the level of consumers’ perceived control, the more useful consumer will perceive intelligent online services to be. Therefore, we propose the following hypothesis:

*Hypothesis 2*: Perceived control positively affects the degree to which consumers perceive intelligent online services to be useful.

### Perceived usefulness and acceptance intention of intelligent services

Perceived usefulness can significantly improve consumers’ perceived enjoyment (Friedrich et al., 2019), positive attitude toward technology use (McLean and Osei-Frimpong, 2019), and intention to accept technology (Venkatesh et al., 2012). The perceived usefulness of e-commerce websites can significantly affect consumers’ online behaviors, such as website usage behaviors (Cheng et al., 2022). In addition, consumers are faced with many uncertainties in the online shopping experience, such as information asymmetries (Shao et al., 2019). If consumers perceive an intelligent online services technology to be useful, they are likely to have a higher degree of trust in the service provider and thus have a higher acceptance intention (Hampton-Sosa and Koufaris, 2005; Cheng et al., 2022). In addition, Friedrich et al. (2019) find that users’ perceived usefulness of intelligent technology has a significant and positive correlation with their intention to use the technology. Following Friedrich et al. (2019), it can be argued that improving consumers’ perceived usefulness of intelligent online services technology may increase their intention to adopt it. Therefore, we propose the following hypothesis:

*Hypothesis 3*: Perceived usefulness positively affects consumers’ acceptance intention of intelligent services.

### Perceived usefulness and sense of power

Sense of power refers to an individual’s perception of their ability to influence others in social relations and asymmetric control of valuable resources (Galinsky et al., 2003). The individual’s sense of power is mainly determined by two factors: individual character and social background (Anderson et al., 2012). In social interactions, sense of power reflects individuals’ influence on the attitudes, behaviors, and results of other individuals in social interactions (Magee and Galinsky, 2008). Previous studies have found that individuals with a higher sense of power tend to have a higher sense of control and lower environmental constraints than those with a lower sense of power (Bandura, 1977). Some studies show that consumers’ sense of power affects their acceptance intention of advertisements (Jin et al., 2014; Wang and Zhang, 2020). In addition, the power approach inhibition theory shows that consumers with a high sense of power are more likely to pay attention to positive information, have more self-confidence, and show positive emotions and disinhibition behaviors in social interactions and choice judgments, while consumers with a low sense of power are more likely to pay attention to risk threat information, have lower self-confidence, and show negative emotions and disinhibition behaviors (Keltner et al., 2003). Especially, the power approach inhibition theory explains the relationship between power sense and self-efficacy. Individuals with high power sense have a higher level of self-efficacy, while individuals with low power sense have a lower level of self-efficacy (Bandura, 1977). In addition, See et al. (2011) find that individuals with a higher sense of power tend to be overconfident and cognitively optimistic and thus overestimate the competence of service providers and give better evaluations. Therefore, this study assumes that individuals with high power are more likely to ignore the risks brought about by technology and have an inflated perception of the usefulness of a technology, while those with low power are excessively concerned with negative information and risks and have a lower perception of the usefulness of that technology. Therefore, we propose the following hypothesis:

*Hypothesis 4*: The higher the consumer’s sense of power, the greater the impact of their perceived control on a technology’s perceived usefulness.

**Figure 1:**
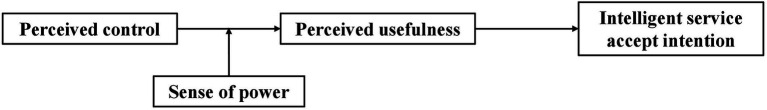
Research model.

According to the research hypotheses proposed above, this study constructs the following theoretical model, as shown in Figure 1.

## Research design

### Experimental design

#### Scenario selection

This study selected an online recommendation intelligent customer service in a travel service app as the experimental setting for the following three reasons. First, in a service such as tourism, young people tend to use intelligent services to complete simple and repetitive tasks (Ivanov et al., 2017), which is consistent with the target group of the experiment. Second, intelligent services require continuous enhancement through the learning and adaptive training capabilities of AI and should not only remain at the technical level of being able to assist human beings but also consider the social attributes of AI (Huang and Rust, 2021b). Intelligent customer service is one of the most effective ways to consider both the technical level of an AI as well as the nature of its social interactions with customers. Third, the most widely used AI technology is the intelligent recommendation system (Chung et al., 2016), which uses an intelligent online customer service system as the experimental setting to ensure that subjects understand the setting.

#### Design and procedures

**Figure 2:**
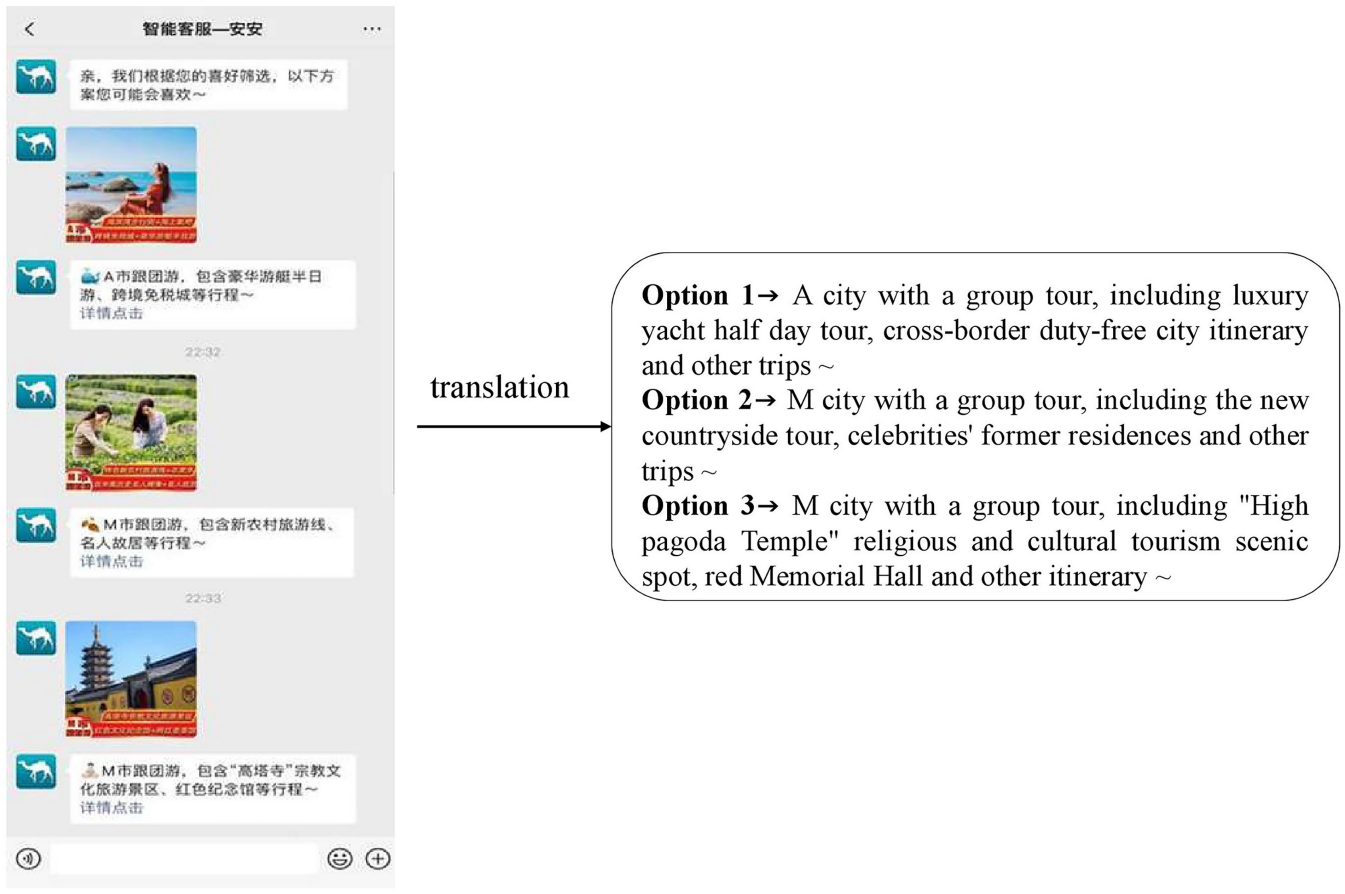
Experimental scenario of the number of recommended options 3. Images source from: m.qunar.com. Reproduced with permission.

**Figure 3:**
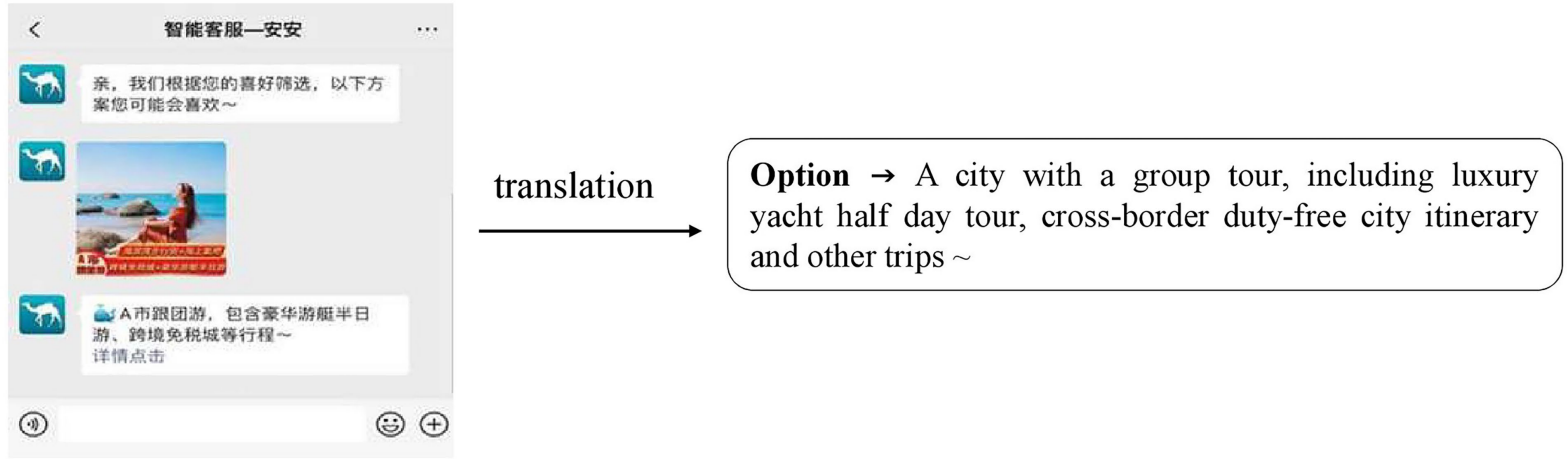
Experimental scenario of the number of recommended options 1. Images sourced from: m.qunar.com. Reproduced with permission.

This study used a group experiment (number of recommended options: 3 *vs*. 1). The “Wenjuanxing” platform was used to design experiments and generate links as well as recruit participants. The experimental questionnaire consisted of five parts. The first part informs the subjects that this is an experiment on memory. The stronger the immersion in reading, the stronger the memory effect, so as to prevent the subjects from guessing the intention of the experiment. The second part described the experimental scenario as “Imagine that after working and studying for a long time, you are taking a long vacation in a few weeks and plan to travel to the domestic coastal city A or M with your friends. After a lot of discussion, everyone has decided to let you make the travel plans, and the *per capita* budget is around 8,000 yuan RMB. However, because there are many scenic spots in City A and City M, which are far away from the urban areas, it will be troublesome to travel freely, so you plan to sign up for a group tour. At this time, you open the commonly used travel app and ask to speak with customer service. An intelligent customer service robot nicknamed “An An” immediately replies with the following message. Please fill in the relevant questions after reading the customer service reply (the number of recommended options 3 is shown in Figure 2, and the number of recommended options 1 is shown in Figure 3).” The third part presented the measurement scales for perceived control, sense of power, perceived usefulness, and acceptance intention. The fourth part requested demographic information, such as the gender, education, and age of participants. The fifth part included the measurement items about the participants’ subjective feelings toward, and familiarity with, the experimental materials. The participants were randomly assigned to one of two experimental groups. First, the participants imagined that they were consulting an intelligent customer service while making the travel group booking plan in the travel app by reading descriptions. Then, they see the intelligent customer service response content corresponding to their experimental group. Finally, participants complete the relevant questions and provide their demographic information. A total of 195 participants participated in the experiment, of which 128 completed the experiment. Specifically, there were 64 samples in the 3-recommended-options group and 64 samples in the 1-recommended-option group. Among the 128 samples, 49 were male, accounting for 38.3%, and 79 were female, accounting for the remaining 61.7%. In terms of age, the participants were mainly young people, concentrated in the two age groups of 16–22 years of age and 22–30 years of age, accounting for 63.6 and 35.7% of the sample, respectively.

### Variable manipulation

This study divides the recommendations of intelligent customer services into 3-option and 1-option schemes. In order to simulate the real online travel ordering scenario, the experiment referred to the tourism product display of the existing online platform. At the same time, in order to reduce the error caused by the subjective bias of the subjects to the recommended content, the options are all either virtual or vague. In addition, in order to eliminate the impact of service satisfaction risk, the scenario provides the requirements and restrictions for seeking solutions, and the matching solutions are given in both experimental scenarios.

### Variable measurement

Perceived control was measured using four items based on a scale following Collier and Sherrell (2010), and modified to better reflect the context of this research. Specific questions include: “In the process of customer service recommendation, I believe I can master the choice of the final plan,” “I feel that I have the right to make a decision in the customer service recommendation program,” “In the customer service recommendation, I feel that my preferences or characteristics affect the content that the customer service recommends to me,” and “When the customer service recommends other solutions (such as recommending restaurants, attractions, etc.), I feel that I have the right to decide.”

Perceived usefulness was measured using four items based on a scale following Venkatesh et al. (2012) and modified to better reflect the context of this research. Specific questions include “Customer service recommendations improve my ability to find the desired solution,” “Customer service recommendations can help me find the most useful information effectively,” “Customer service recommendations provide quick answers to my questions,” and “Overall, I found customer service recommendations useful.”

Acceptance intention was measured using three items based on a scale following Venkatesh et al. (2012) and modified to better reflect the context of this research. Specific questions include “I will consider using customer service recommendations for future tour bookings,” “I will also use customer service recommendations for future tour bookings,” and “I will also use customer service recommendations if other problems arise during the trip (such as finding the right hotel).”

Sense of power was measured using four items based on a scale following Anderson et al. (2012) and modified to better reflect the context of this research. Specific questions include “In my daily life, I can make others listen to what I say,” “In my daily life, I can often make others do what I want,” “In my daily life, I think I have a lot of power,” and “In my daily life, I can make my own decisions as long as I like.”

The above items were measured using a 7-level Likert scale, where 1 means “strongly disagree” and 7 means “strongly agree.” In addition, all measurement items were translated from Chinese to English. Comparing the questionnaires before and after translation, no semantic differences were found.

## Data analysis

### Reliability and validity test

In this study, SPSS24.0 was used to test the reliability and validity of the experimental data. As shown in Table 1, the Cronbach’s α for all variables is above 0.7, thus suggesting that the internal consistency of each construct is high. In addition, confirmatory factor analysis is used to test the combination reliability (CR). As shown in Table 1, the combination reliability of each construct is higher than 0.7, thus indicating that the combination reliability of each construct is good. Therefore, each construct in the model has good reliability.

**Table 1:** Test for reliability and validity.

Variables	Items	Mean	SD	FL	Alpha	CR	AVE
Perceived control (PC)	PC1	4.86	1.332	0.886	0.907	0.936	0.785
	PC2	4.90	1.229	0.912			
	PC3	4.99	1.181	0.904			
	PC4	5.02	1.072	0.842			
Perceived usefulness (PU)	PU1	4.98	0.887	0.858	0.874	0.914	0.727
	PU2	4.96	0.942	0.860			
	PU3	5.01	0.918	0.827			
	PU4	4.98	0.951	0.865			
Acceptance intention (AI)	AI 1	4.88	0.980	0.885	0.819	0.895	0.741
	AI 2	4.69	1.010	0.888			
	AI 3	4.73	1.133	0.806			
Sense of power (SP)	SP1	4.55	1.078	0.769	0.709	0.861	0.610
	SP2	4.21	1.113	0.865			
	SP3	4.20	1.211	0.722			
	SP4	5.19	1.107	0.759			

For the validity of the scale problem, the Kaiser-Meyer-Olkin (KMO) of the scale used in this research is 0.935, which is above 0.6, and the significance of Bartley’s spherical test is less than 0.05, which is suitable for exploratory factor analysis. In addition, for confirmatory factor analysis, as shown in Table 1, the standard factor load (FL) of each item is greater than 0.5, which suggests that the overall convergence validity is good. The value of the average variance extracted (AVE) of all variables is higher than 0.5, which indicates that the model has sufficient discriminant validity.

### Correlation analysis

As shown in Table 2, the Pearson’s correlation coefficients between perceived control and perceived usefulness and acceptance intention are 0.806 and 0.678, respectively, thus suggesting that there are positive correlations between perceived control and the other two variables. In addition, the Pearson’s correlation coefficient between perceived usefulness and acceptance intention is 0.790, which shows that there is a positive correlation between these two variables. Finally, the Pearson’s correlation coefficient between perceived control and perceived usefulness and sense of power are 0.488 and 0.542, respectively, thus indicating that there are positive correlations between these two variables and sense of power.

**Table 2:** Correlation coefficient matrix.

Variables	PC	PU	AI	SP
PC	0.886			
PU	0.806	0.852		
AI	0.678	0.790	0.860	
SP	0.488	0.542	0.533	0.781

The value on the diagonal is the square root of AVE, and other data are the correlation coefficients between the corresponding variables.

### Manipulation check

The manipulation of perceived control was examined using an independent-samples t-test. As shown in Table 3, M_low perceived control_ = 3.079 with a standard deviation (SD) of 0.797, and M_high perceived control_ = 5.727 with an SD of 0.640. *p* < 0.001 and the result is significant, which indicates that there is a significant difference in the level of perceived control between the two groups of subjects. Thus, the manipulation of perceived control is supported.

**Table 3:** Manipulation check results for perceived control.

Group	N	Mean	SD	SE
Low perceived control	64	4.156	0.797	0.099
High perceived control	64	5.727	0.640	0.080

### Hypothesis test

#### Main effect testing

**Figure 4:**
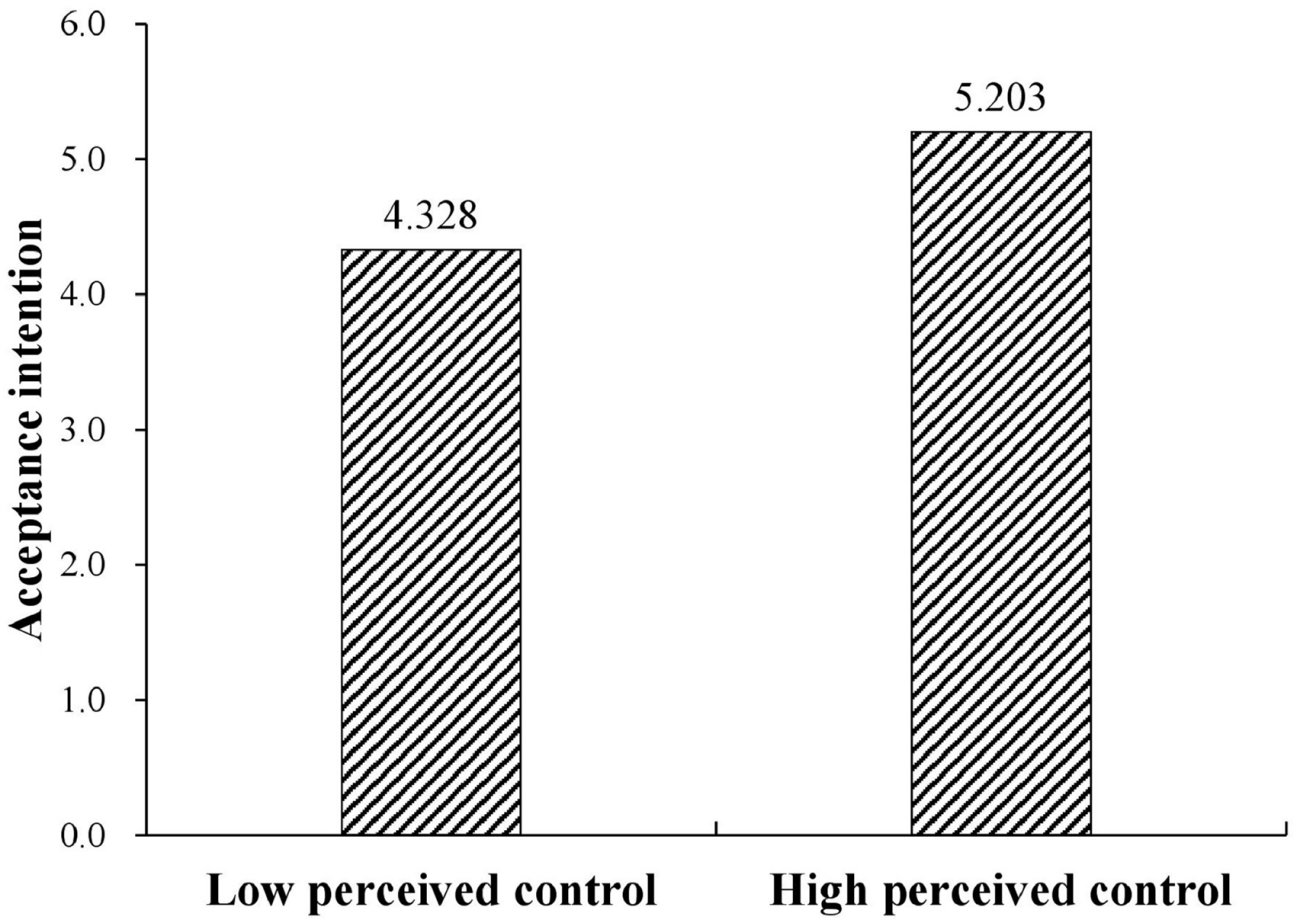
Acceptance intention under different levels of perceived control.

This study uses the independent-samples t-test to verify the influence of perceived control on consumers’ acceptance intention of intelligent services. Figure 4 shows consumers’ acceptance intentions of intelligent online services under different levels of perceived control. The results show that the M_high perceived control_ = 5.203 with an SD of 0.868, and the M_low perceived control_ = 4.328 with an SD of 0.684. The effect of the high perceived control group on acceptance intention was significantly higher than that of the low perceived control group (*p* < 0.001, t (126) = 6.335). Therefore, H1 is supported.

#### Core path testing

This research uses the PROCESS plug-in of SPSS23.0 to test the relationship between perceived control, perceived usefulness, and consumer acceptance intention based on regression analysis and the bootstrapping method. In the present study, 5,000 samples are used in the bootstrapping analysis, the sampling method is the non-parametric percentile method of bias-corrected correction, and the 95% confidence interval is constructed for testing.

As shown in Table 4, for the correlation path between perceived control and perceived usefulness, T = 15.173, β = 0.593, and *p* < 0.001, thus indicating that perceived control has a significant and positive impact on perceived usefulness. In addition, for the correlation path between perceived usefulness and acceptance intention, T = 14.449, β = 0.895, and *p* < 0.001, which confirms that perceived usefulness has a significant and positive influence on acceptance intention. Therefore, H2 and H3 are supported.

**Table 4:** Core path testing.

Paths	Coefficients	SD	T	P
Perceived control –> Perceived usefulness	0.593	0.039	15.173	0.000
Perceived usefulness –> Acceptance intention	0.895	0.062	14.449	0.000
Perceived control –> Perceived usefulness –> Acceptance intention	0.784	0.104	7.550	0.000

In addition, this study takes perceived control as an independent variable, perceived usefulness as a mediating variable, and acceptance intention as a dependent variable to verify whether perceived usefulness plays a mediating role between perceived control and acceptance intention. As shown in Table 4, the confidence interval of the indirect influence of perceived control on acceptance intention is [0.313, 0.631], excluding zero (*p* < 0.001), which indicates that the mediation effect is significant with a value of 0.465. After controlling the perceived usefulness of the mediator variable, the direct effect of perceived control on acceptance intention is not significant (β = 0.102), and the confidence interval is [−0.049, 0.253], including zero. Therefore, perceived usefulness plays a significant mediating role in the influence of perceived control on consumers’ acceptance intention.

#### Moderating effect testing

This study used SPSS regression analysis to examine the moderating effect of sense of power between perceived control and perceived usefulness. Sense of power is set as a classified variable to verify that it plays a moderating role in the relationship between perceived control and perceived usefulness.

**Figure 5:**
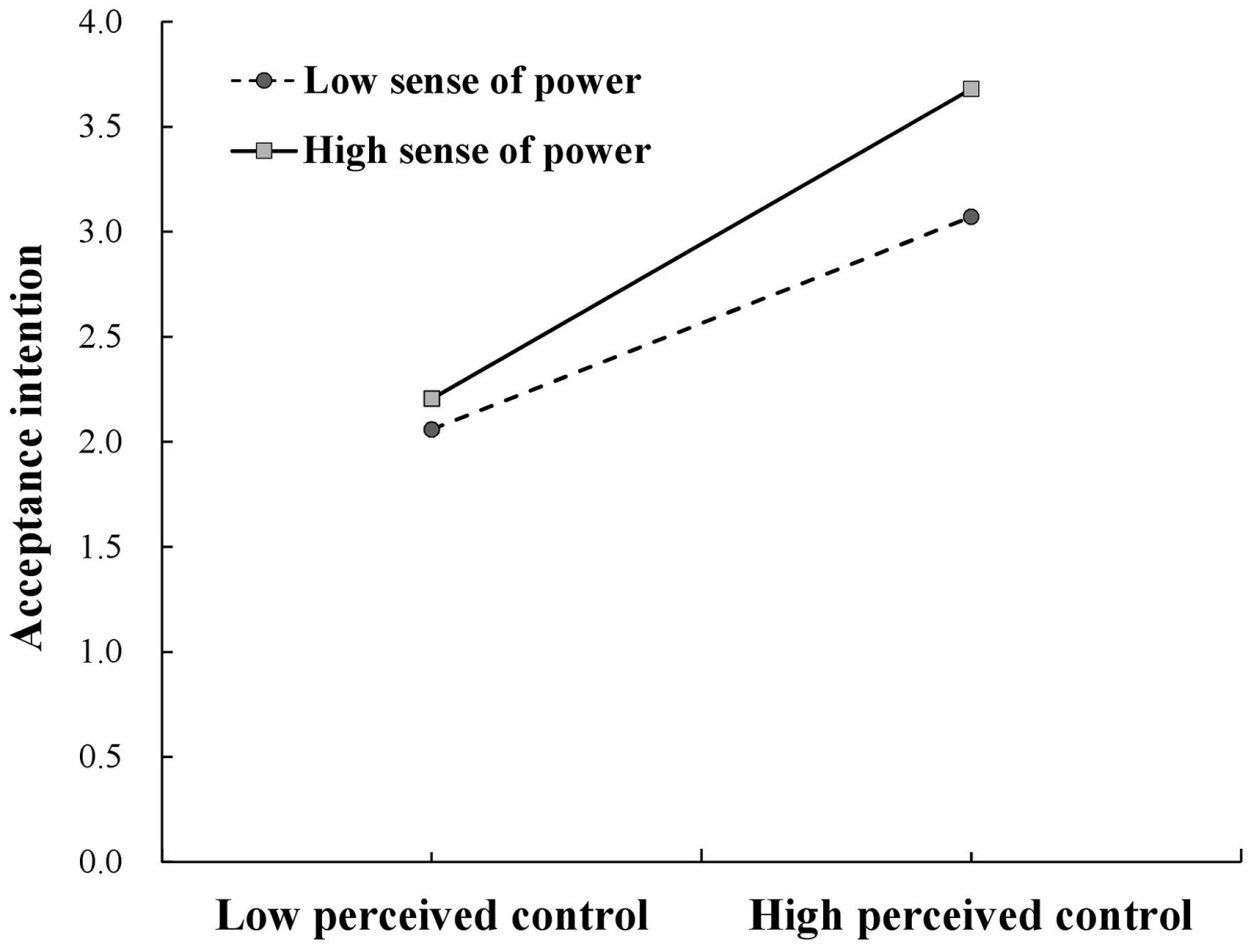
Moderating effect test of sense of power.

The test results show that the interaction terms of perceived control and sense of power have a significant influence on perceived usefulness (β = 0.231, *p* < 0.05, *R*^2^ = 0.672). Figure 5 further tests the moderating effect of sense of power. According to the results, When sense of power is low, compared with low perceived control, consumers with high perceived control have stronger acceptance intention (β = 0.506, *p* < 0.001, 95% confidence interval is [0.412, 0.599], excluding zero). When the sense of power is high, compared with low perceived control, consumers with high perceived control have stronger acceptance intention (β = 0.737, *p* < 0.001, 95% confidence interval is [0.562, 0.911], excluding zero). Therefore, sense of power moderates the relationship between perceived control and perceived usefulness, and thus H4 is supported.

## General discussion

### Research conclusion

Based on the technology acceptance model, this study investigated the influence of perceived control on consumers’ acceptance intention of intelligent online services and examined the moderating effect of consumers’ sense of power.

There are three findings in this study. First, consumers’ perceived control affects their acceptance intention of intelligent services; the higher consumers’ perceived control, the stronger their acceptance intention of intelligent online services will be. This also further illustrates the effect of consumers’ perceived control over recommendation depth on the recommendation effect of recommendation agents, further developing the research conclusions of Ghasemaghaei (2020).

Second, consumers’ perceived control affects their perceived usefulness of intelligent online services as well as their acceptance intention of intelligent services. This further validates similar studies that the number of consumer choices can affect consumers’ acceptance intentions (Hui and Bateson, 1991).

Third, consumers’ sense of power moderates the influence of perceived control on perceived usefulness. When consumers’ sense of power is high, the influence of consumers’ perceived control on the usefulness of intelligent online services is more significant. This further develops the research conclusion of Wang and Zhang (2020).

### Theoretical implications

This study makes several theoretical contributions. First, this study investigates the influence of perceived control on consumers’ acceptance intention of intelligent online services from the perspective of social interaction, which enriches the research on intelligent online services. Prior studies have focused on the interactive perspective of consumer perception and specific intelligent online services scenarios and investigate the influence of the characteristics of such services in terms of their anthropomorphic behaviors, task-orientation, and personalized interaction on consumers’ attitudes and behavioral intentions. However, there are few studies on the influence of consumer-perceived control on the acceptance intention of intelligent services. In practice, overemphasizing intelligent online services may destroy consumers’ sense of autonomy and directly affect their acceptance intention. Therefore, this study focuses on the perceived control and sense of power that arise in the process of social interaction to develop a new direction in the research on intelligent customer service.

Second, this study introduces consumers’ sense of power, which defines a new boundary condition, as a moderating factor in the field of online intelligent customer service research. Previous studies mainly examine the relationship between service providers and users, while this study proposes and verifies the moderating effect of consumers’ sense of power based on an actual interactive setting recommended by an intelligent customer service in a travel app. The conclusions deepen our knowledge of the moderating factors of intelligent services.

Third, this study expands the potential applications of the technology acceptance model. Based on this model, this study proposes and verifies that perceived usefulness plays a mediating role in the influence of consumer-perceived control on intelligent online services acceptance intention. Therefore, this study introduces perceived control into the research on technology acceptance, which expands the potential applications of this theory.

### Practical implications

The current research is of great significance to online enterprises. First, this study causes online merchants to rethink and attach renewed importance to results- and process-oriented service goals. The results show that the number of recommended options in intelligent online services affects consumers’ perceived control, which then affects their acceptance intention of intelligent services. Therefore, online merchants should consider whether to reduce consumers’ perceived control while providing them with faster and more convenient services. When consumers’ perceived control is low, the technology originally aimed at optimizing services may not serve its intended purpose, thus compromising the connection between consumers and enterprises.

Second, this study provides a reference for online merchants to use in formulating intelligent product recommendation strategies. The results of this study show that consumers’ sense of power moderates the influence of their perceived control on the perceived usefulness of intelligent services. When consumers have a greater sense of power, consumers’ perceived control has a greater impact on the perceived usefulness of intelligent services. Therefore, online merchants should market “smart” products according to consumers’ sense of power and divide the target markets of such products in a targeted manner. Specifically, in the early stages of the lifecycle of smart products, enterprises should take consumers with a high sense of power as the target market because such consumers have higher acceptance intentions with regard to smart products and are more likely to accept them.

### Limitations and further research

Although the findings of this study are valid and valuable, there are still some limitations that provide directions for future research. First, this study explored the influence of perceived control on consumers’ acceptance intention through a scenario-based experiment. We structured our research in this way because it is a causal exploratory study of consumer behavior, which requires strict control of many factors and must ensure high internal validity. Future studies can collaborate with online merchants and conduct field studies to further verify the relationship between perceived control and consumers’ acceptance intention.

Second, this study used the intelligent customer service recommendations of a travel app as the experimental scenario because online intelligent customer service recommendation services are the most widely used intelligent online services technologies at present and young people tend to use intelligent online services to fulfill related needs in the field of travel services. Future research can be extended to different experimental scenarios to verify and improve the robustness of the research conclusions.

Third, this study only examines the influence of perceived control on perceived usefulness, and then on consumers’ acceptance intention. Future studies can examine the influence of consumers’ acceptance intention of intelligent online services from the aspects of perceived ease of use and privacy concerns.

Finally, this study focuses on the adolescents’ acceptance of intelligent online services, and the subjects in the research experiment are mainly concentrated in adolescents. Future research can further verify the applicability of this research model in other age groups, and compare the interaction of different age groups on consumers’ acceptance intention of intelligent online services.

## Data availability statement

The raw data supporting the conclusions of this article will be made available by the authors, without undue reservation.

## Ethics statement

Ethical review and approval was not required for the study on human participants in accordance with the local legislation and institutional requirements. The patients/participants provided their written informed consent to participate in this study.

## Author contributions

YY, WF, and YL contributed to the conceptualization, methodology, statistical analysis, data curation, and writing. BS and WF contributed to the revision, investigation, supervision, funding acquisition, and project administration. All authors contributed to the article and approved the submitted version.

## Funding

We acknowledge the financial support from the National Natural Science Foundation of China (grant nos.: 72110107002 and 71974021), in part by the National Social Science Foundation of China (grant nos.: 21BGL246).

## Conflict of interest

The authors declare that the research was conducted in the absence of any commercial or financial relationships that could be construed as a potential conflict of interest.

The handling editor YC declared a shared affiliation with the authors WF and BS at the time of review.

## Publisher’s note

All claims expressed in this article are solely those of the authors and do not necessarily represent those of their affiliated organizations, or those of the publisher, the editors and the reviewers. Any product that may be evaluated in this article, or claim that may be made by its manufacturer, is not guaranteed or endorsed by the publisher.
